# Perceptions of Medical Cannabis Packaging and Labeling among Middle-Aged and Older Canadians

**DOI:** 10.1093/geroni/igab046.3720

**Published:** 2021-12-17

**Authors:** Melissa O'Connor, Vanessa Christiuk, Megan Pedersen

**Affiliations:** 1 North Dakota State University, Moorhead, Minnesota, United States; 2 Eloski Health Centre, Fargo, North Dakota, United States; 3 North Dakota State University, Fargo, North Dakota, United States

## Abstract

The use of cannabis for therapeutic purposes is becoming more popular in many countries, including the United States and Canada. In Canada, middle-aged and older adults make up the largest proportion of medical cannabis users. Canadian legislation mandates that medical cannabis be packaged in plain-looking containers with small labels, childproof caps, and required health warnings. This is meant to standardize the way cannabis products are distributed, as well as protect children from accidental ingestion. However, there is limited research on how these regulations affect cannabis users over age 45. In the present study, residents of Winnipeg, Manitoba, Canada aged 45 and older (n=40) were surveyed regarding their experiences with medical cannabis packaging and labeling. Half of the participants (50%) felt they had a hard time opening their medical cannabis container. A majority (60%) thought having an easy-open lid would be helpful. Most participants (78%) reported experiencing difficulties reading the label on their container, and 75% thought it would be helpful to have a printout of the label in a larger font. In addition, 89% of participants who took more than one kind of medical cannabis favored a symbol on their medication bottle that would indicate the type of medical cannabis contained inside. Implications for policy makers and future research are discussed.

